# Volatile organic compounds in *Solanum lycopersicum* leaves and their roles in plant protection

**DOI:** 10.1093/hr/uhaf181

**Published:** 2025-07-16

**Authors:** Simona Gargiulo, Michelina Ruocco, Francesco Loreto, Luigi Faino, Maurilia Maria Monti

**Affiliations:** Department of Environmental Biology, University of Rome Sapienza, Piazzale Aldo Moro 5, 00185 - Rome (RM), Italy; Institute for Sustainable Plant Protection (IPSP), National Research Council (CNR), P.le E. Fermi 1, 80055 - Portici (NA), Italy; Institute for Sustainable Plant Protection (IPSP), National Research Council (CNR), P.le E. Fermi 1, 80055 - Portici (NA), Italy; Department of Biology, University of Naples Federico II, Naples, Italy; Department of Environmental Biology, University of Rome Sapienza, Piazzale Aldo Moro 5, 00185 - Rome (RM), Italy; Institute for Sustainable Plant Protection (IPSP), National Research Council (CNR), P.le E. Fermi 1, 80055 - Portici (NA), Italy

## Abstract

Tomato (*Solanum lycopersicum* L.) is a species of high economic value, an essential food source, and a model organism for both applied and basic research in crop science. Tomato plants also produce and emit a wide variety of volatile organic compounds (VOCs), which are thought to play a prominent role in multitrophic interactions. This review aims to provide a comprehensive overview of the extensive literature about tomato VOCs emitted by leaves. We explored the role of VOCs in the interactions of tomato plants with the environment, focusing on VOCs that provide plant protection against herbivores, pathogen vectors, pathogens, and abiotic stresses. VOC functions in plant–plant communication and defence are less known, but new evidence is now being collected showing that VOCs sent by plants can inform neighbour plants about impending stresses. Overall, improved knowledge on VOC biochemistry and functions may soon allow their use for sustainable protection practices of tomato crops. Remaining gaps and promising areas for future research are also examined.

## Introduction

All living organisms emit volatile organic compounds (VOCs), including alcohols, aliphatic and aromatic hydrocarbons, aldehydes, ketones, ethers, and acids. VOCs are mostly lipophilic molecules, with small molecular weight (50–200 Da) and high vapour pressure at ambient temperature [1]. Plants can emit up to 1700 VOCs from all their organs. VOCs are the main components of the scent of flowers and the smell of leaves and fruits*.* VOCs can be synthesized constitutively, either throughout the plant life cycle or at specific growth stages, or their production can be induced by stress conditions [2–4], suggesting specific defensive functions against abiotic and biotic stresses. For example, in response to abiotic stresses, VOCs may stabilize cell membranes [5]; after an injury, they may convey systemic acquired resistance (SAR) to different parts of the plants [4, 6]; and thanks to their antimicrobial effect [7], VOCs may reduce pathogen development [8]. There is also substantial evidence supporting the crucial role of VOCs in complex interactions between plants and other organisms, especially insects or fungi [9–11].

Moreover, VOCs are likely involved in plant–plant interactions and have a role in indirect plant defence. The first evidence of herbivore resistance induction in healthy plants adjacent to attacked ones was reported 40 years ago [12], and since then, VOC-driven plant–plant interaction has been repeatedly observed [13–19]. Numerous studies have proven that some VOCs activate transcription factors, triggering changes in the expression of genes involved in plant defence [20–23]. However, how plants can perceive the VOCs emitted by neighbouring stressed plants remains unclear [5, 22].

Much of the research on plant VOCs has focused on crop plants to explore their potential applications in enhancing resistance against climate change-associated stresses (e.g. heat spells, droughts, extreme events, pests, and pathogen spread) [6, 17, 24–26]. Tomato (*Solanum lycopersicum* L.) is a key crop worldwide, both in terms of production and consumption (186 million tons of tomatoes were produced globally in 2022) [27, 28]. Several features (such as a short life cycle, a relatively small genome, diploidy, self-pollination, and easy propagation through vegetative explants [29]) make tomato an important model plant suitable for biotechnology applications. As a model species, the tomato plant volatilome has been extensively explored not only for its organoleptic relevance in fruits [30] but also for its role in plant–environment interactions. However, the presence of VOC storage organs on the leaf surfaces, such as trichomes, complicates the investigation of VOC production and emission from tomato vegetative tissues.

Mechanical damage, indeed, may significantly affect the amount of VOCs detected during measurements (usually ranging between 5 and 20 ng m^−2^ s^−1^ [31]), and distinguishing VOCs that are ‘actively’ emitted from those released due to rough handling becomes particularly challenging in tomato. Among the most common and efficient VOC sampling methods in tomato plants is dynamic headspace collection, with plants enclosed in glass bell chambers under a constant airflow, without any leaf manipulation. The volatiles are either trapped into air sampling cartridges for successive analysis by gas chromatography–mass spectrometry (GC–MS) or directly sampled by proton transfer reaction–time of flight–mass spectrometry. This latter method allows real-time VOC sampling in a nondisruptive way and with unprecedented accuracy (ppt level). Detailed information regarding the most common VOC sampling and detection methods for *S. lycopersicum* is reported in Table S1.

The following paragraphs will review current knowledge on the role of VOCs emitted by tomato leaves particularly focusing on direct and indirect plant protection, and how these aspects vary depending on the plant genotype, and on current and potential applications of VOCs in tomato crops.

## VOC biosynthesis and gene regulation

**Figure 1:**
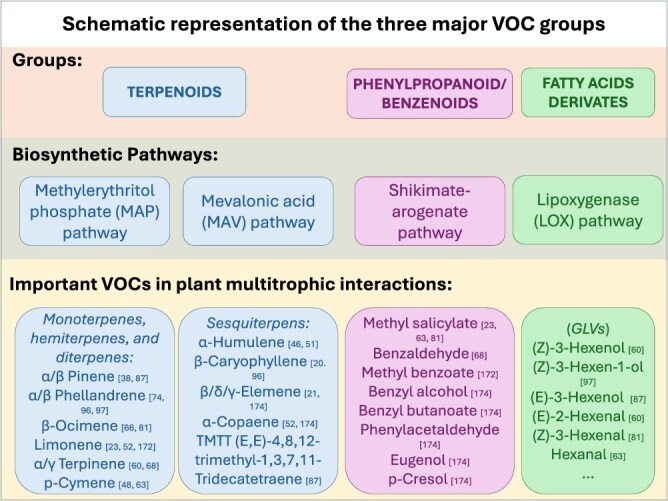
Overview of major VOCs classified by chemical structure and biosynthetic pathways.

Plant VOCs may be categorized in three major groups, based on their biosynthetic origin: terpenoids, phenylpropanoids/benzenoids, and fatty acid derivatives (Fig. 1).

The group of terpenoids is the largest, accounting for more than 500 compounds, comprising two major categories of mono and sesquiterpenes. Terpenoids are compounds derived from isoprene units, playing essential roles in physiological and biochemical processes such as photosynthesis, electron transport, developmental regulation, and membrane architecture. Many terpenoids are specialized to defend plants against biotic and abiotic stress, and to attract beneficial soil microorganisms [32]. These compounds are synthesized from two precursors, isopentenyl pyrophosphate (IPP) and dimethylallyl pyrophosphate (DMAPP), which constitute the building blocks of terpenoids, via two biosynthetic pathways: mevalonic acid pathway (MAV) and methylerythritol phosphate pathway (MEP). The MAV is localized mainly in the cytoplasm and contributes to the synthesis of sesquiterpenes (C15) [33]. In this pathway, acetyl-CoA is condensed and reduced through enzymes like acetoacetyl-CoA thiolase, 3-hydroxy-3-methylglutaryl-CoA synthase/reductase to produce mevalonate, which is subsequently phosphorylated and decarboxylated by mevalonate kinase, phosphomevalonate kinase, and mevalonate-5-diphosphate decarboxylase to form IPP, the most immediate isoprenoid precursor. The MEP pathway is localized mainly into the chloroplast and leads to the synthesis of hemiterpenes, monoterpenes, and diterpenes. In the MEP pathway, pyruvate and glyceraldehyde-3-phosphate are condensed to produce 1-deoxy-d-xylulose 5-phosphate by DXS (synthase), subsequently reduced to MEP by DXR (reductase). Further enzymatic steps convert MEP into IPP and DMAPP.

Both pathways provide substrates to terpene synthases (TPSs), a large family of enzymes able to synthesize the hundreds of terpenoids available in nature. In fact, even a single amino acid change in a TPS can induce a modification of the mixture of synthetized terpenes [32]. Tomato plants produce a remarkable variety of terpenes, particularly in trichomes present on leaves, stems, young fruits, and part of the flowers. However, they do not contribute strongly to the fruit aroma, and their role is considered mainly defensive [34]. Recent studies reveal that the tomato genome contains 34 functional TPS genes, with their catalytic activities, expression patterns, and subcellular localisations fully characterized, making the tomato TPS family the first to be completely studied [35]. In tomato, the upregulation of TPS enzymes induced under biotic and abiotic stress has been widely demonstrated, further confirming the terpenoids’ active role as a plant defence mechanism. For example, the TPS *MTS1* is upregulated after bacterial infection and correlated with the production of several hemiterpenes (e.g. linalool or α-terpineol) [36], while *TPS5* (a monoterpene synthase) and *TPS12* (a β-caryophyllene/α-humulene synthase) are associated with herbivore direct feeding and elicitor presence [21]. Upregulation of the *TPS12* has also been associated with tomato heat stress response together with β-phellandrene synthase *TPS20* [37].

Phenylpropanoids/benzenoids (PBs), the second largest group of plant VOCs, are volatile aromatic compounds that share the aromatic amino acid phenylalanine as a precursor. They can be grouped into benzenoids (C6–C1) (e.g. benzyl alcohol and benzaldehyde), phenylpropanoid-related compounds (C6–C2) (e.g. phenylacetaldehyde and 2-phenylethanol), phenylpropanoids (C6–C3) (e.g. eugenol), and polyketides, compounds synthesized by type III polyketide synthases (PKS III) from cinnamyl-CoA and malonyl-CoA [37]. Their biosynthesis begins with the deamination of phenylalanine by phenylalanine ammonia lyase (PAL), producing trans-cinnamic acid (t-CA), a key precursor. Further methylation or acylation by specific enzymes generates diverse PBs. The volatile PBs are essential for the flower scent in many plants, and they have a relevant ecological role (e.g. phenylacetaldehyde is effective against herbivore attacks, probably acting as repellents [38, 39], benzyl alcohol and benzaldehyde attract parasitoids in rice [40], and methyl salicylate [MeSa] with its multiple defensive and signalling role belongs to this group [41]). In tomato, recently, transcriptomic and metabolomic analyses identified key genes and transcription factors regulating this complex pathway. The MYB–bHLH–WD40 complex, particularly MYB transcription factors, play a significant role in phenylpropanoid regulation, influencing the expression of genes like PAL, 4-coumarate:CoA ligase (4CL), and cinnamate-4-hydroxylase (C4H). These genes catalyse critical steps in phenylpropanoid metabolism, leading to the synthesis of volatile and nonvolatile phenylpropanoid derivatives, including lignin precursors, flavonoids, and phenolic compounds [42].

The third group comprises fatty acid derivatives, originating from catabolism of C18 unsaturated fatty acids as linolenic and linoleic acids via lipoxygenase (LOX) activity. They include the phytohormone methyl jasmonate (MeJA) and the green leaf volatiles (GLV), well-known bioactive compounds consisting of six-carbon (C6) aldehydes, alcohols, and their esters [3, 42]. This pathway involves two main steps: first, lipoxygenases (13-LOX and 9-LOX) oxygenate fatty acids to form 13- or 9-hydroperoxides. Second, hydroperoxide lyases (13-HPL and 9-HPL) cleave these hydroperoxides into oxoacids and volatile aldehydes. These aldehydes can be further converted into alcohols by alcohol dehydrogenases (ADH) [30]. In tomato, 14 LOXs have been identified divided into 13-LOX (*SlLOX3, SlLOX4, SlLOX10, SlLOX11, SlLOX12*) and 9-LOX (*SlLOX1, SlLOX2, SlLOX5, SlLOX6, SlLOX7, SlLOX8, SlLOX9, SlLOX13, SlLOX14*) [43]. In the literature, many examples have reported the involvement of those genes in tomato plant response to abiotic or biotic stress, and correlated with GLV emissions and MeJA signalling [36, 44–46].

## The role of VOCs in stress response: insights from tomato wild relatives

Many studies have explored the spectrum of VOC emission in tomato cultivars and in their wild relatives. Bleeker *et al.* [47] investigated five tomato cultivars, comparing them to 16 wild tomato accessions of *Solanum pennellii*, *Solanum habrochaites*, and *Solanum peruvianum*. The study revealed that the whitefly *Bemisia tabaci* (Gennadius), an important pest and vector of many viruses affecting Solanaceae species, consistently preferred all cultivated varieties. The volatilomes of wild genotypes were characterized by massive emission of the sesquiterpenes curcumene and zingiberene, along with high emission of γ-terpinene and *p*-cymene (Table S2), compounds likely acting as insect repellents. Other studies confirmed that among the most noteworthy VOCs that are strongly emitted by the *S. lycopersicum* ancestors, making them repellent especially for the whitefly, there is zingiberene, its stereoisomer 7-epizingiberene, or its derivative *R*-curcumene [48–50]. Paudel *et al.* [51] displayed a more complex situation, suggesting a reduction of constitutive defences alongside an increase in inducible ones in domesticated tomato. This study emphasized a distinct clustering of the volatilomes of the three analysed lines (*S. lycopersicum* cv. Better Boy, *S. lycopersicum* var. *cerasiforme*, and *Solanum pimpinellifolium* LA 2093) under attack by the herbivore *Helicoverpa zea* (Boddie)*.* Notably, the Better Boy cultivar exhibited a greater induction of monoterpene emissions (β-phellandrene, 2-carene, α-terpinene, and *p*-cymene, Table S2) potentially serving as attractants for parasitoids. Arafa *et al.* [52] focused on the behaviour of three wild late blight-resistant accessions of *S. habrochaites*, each harbouring distinct resistance genes or genomic regions [33, 34]. These were compared with the susceptible genotypes of *S. lycopersicum* cv. Super Strain B and *S. pimpinellifolium* (LA0375) following infection with *Phytophthora infestans* (Mont.) de Bary*.* The resistant genotypes showed clear differences in trichome types and density, as well as in the production of secondary metabolites and VOCs. Specifically, only the resistant genotypes produced the terpenes β-caryophyllene and geranyl-α-terpinene, the sesquiterpenoids isocurcumenol and ledene oxide, and the organic acid 9-*cis*-retinoic acid (Tables S2–S4), all of which appeared to inhibit the pathogen growth during in vitro and in vivo tests conducted using leaf extracts from resistant plants [52].

The process of domestication, which has led to the development of new species and cultivars with desirable traits tailored to diverse market needs, has often resulted in a reduced capacity for plant defences. This reduction is one of the primary drawbacks of the domestication syndrome [53–57]. VOCs are among the stress-related beneficial compounds that might have been adversely affected by tomato domestication, as they represent an unnecessary carbon cost in relatively stress-free agricultural conditions. All these examples underscore the significant genetic influence on VOC profiles, even at the intraspecific level, and highlight their importance in determining stress resistance in tomato cultivars and accessions. Beyond comparisons between wild ancestors and modern cultivars of *S. lycopersicum*, numerous studies have investigated the volatilome of tomato cultivars in relation to their ability to withstand stresses [50, 58, 59]. The results of these investigations are presented in the following paragraphs.

## Tomato VOCs in the interaction with other organisms

**Figure 2:**
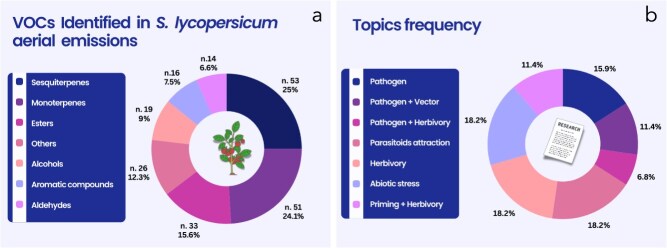
Pie chart showing families of VOCs emitted by *S. lycopersicum* plants (a); Pie chart showing the frequency of *S. lycopersicum* VOC studies (between 1993 and 2024) in relation to stress factors and interaction with other organisms (b).

**Figure 3:**
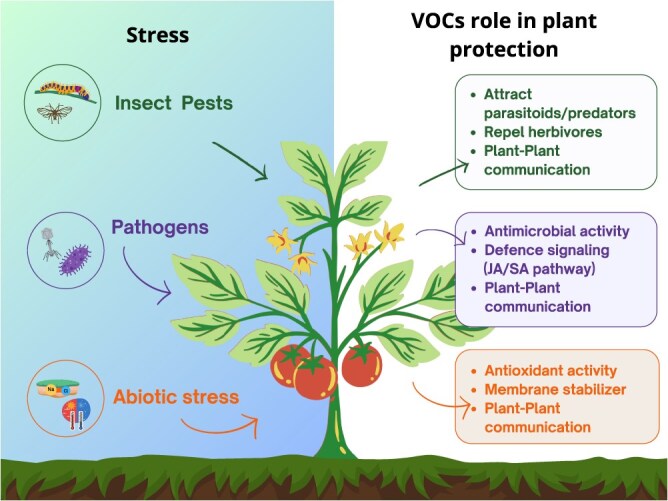
Scheme of the multiple aboveground interaction involving VOC production in tomato plants in response to stressors and their defence-related roles.

We have analysed 44 research studies published between 1993 and 2024, reporting data on VOCs emitted by the aerial vegetative tissues of *S. lycopersicum* (leaves and stems), along with their biochemical classification (Fig. 2a; Tables S2–S4). Investigating VOCs emitted by tomatoes led to the identification of 212 molecules, half of which are terpenes (53 sesquiterpenes and 51 monoterpenes). Emission rates and blends are strongly influenced by different conditions, such as abiotic and biotic stress factors, or rough handling, causing rupture of glandular trichomes serving as VOC reservoirs. Details on tomato VOC emissions are shown in Tables S2–S4. The most recurrent topics include interactions between plants, the environment, and insects (parasitoid attraction: 18.2%; herbivory: 18.2%; abiotic stress: 18.2%). Interactions between tomato plants and pathogens (pathogen: 15.9%) are also frequently studied, often with a focus on pathogen–vector interactions or combined stress scenarios (pathogen + vector: 11.4%; pathogen + herbivory: 6.8%). Despite the extensive literature and the great potential of VOCs in plant–plant communication and priming, their role in tomatoes was addressed in only 11.4% of the studies analysed (Fig. 2b). The complex network of aboveground interactions triggered in tomato plants by various stressors, highlighting the role of volatile organic compounds (VOCs) in mediating defence responses is shown in Fig. 3.

## Tomato VOC emissions in the interaction with insects

Insects and plants have deep and intricate relationships, with insects playing both detrimental (e.g. by feeding on plants) or beneficial roles (e.g. by improving pollination or defending plants against pests). Tomato plants, in particular, can be attacked by a plethora of herbivore insects. Insect feeding causes a primary emission of constitutive VOCs, due to tissue disruption, trichome damage, and cell membrane rupture. This is followed by secondary emissions triggered by insect oral secretions, ovipositional fluids, saliva, secretions from ventral eversible glands, waste products, and herbivore endosymbionts, leading to de novo VOC biosynthesis [60, 61]. Notably, the upregulation of genes encoding TPSs and SlLOXs has been linked to increased VOC emissions and enhanced plant responses to pest attacks [21, 39, 46].

To improve bio-control strategies, numerous studies have investigated whether those insect-induced VOCs are able to ‘recruit’ external allies, such as insect predators and parasitoids, thereby triggering multitrophic interactions to the benefit of the plant. Sasso *et al.* [62] proved that the aphid parasitoid *Aphidius ervii* Haliday was significantly more attracted to tomato plants infested with aphids compared to healthy plants. In a subsequent study, three VOCs, (8*S*,9*R*) -(*E*)-caryophyllene, methyl-salicylate, and (*Z*)-3-hexen-1-ol, were found to elicit a significant electrophysiological response in the antennae of *A. ervii* [63]. Further studies confirmed the attractiveness of *A. ervii* to (*Z*)-3-hexen-1-ol [64–66]. In another case, infestation of the leaf-chewer *Spodoptera littoralis* (Boisduval) induced the emissions of terpenes such as ꞵ-phellandrene, *p*-cymene, 3-carene, limonene, and caryophyllene, increasing the attractiveness of the natural enemy *Trichogramma chilonis* Ishii in *S. lycopersicum* cv. Shaktiman [58]. Ayelo *et al.* [67] found that tomato plants infested by *Trialeurodes vaporariorum* (Westwood) emitted high levels of 3-carene, β-ocimene, β-myrcene, and α-phellandrene, which attracted the parasitoid *Encarsia formosa* Gahan. The same authors, studying a different tritrophic system, reported that the generalist predator *Nesidiocoris tenuis* Reuter responded to a different VOC blend, characterized by a high level of α-pinene and lack of β-myrcene*.* The addition of β-phellandrene reduced attractiveness, while addition of *E*-β-caryophyllene even repelled the predator [68]. Besides terpenes, the well-known bioactive compound MeSa may also attract predators. In fact, mutant tomato plants unable to produce MeSa, when attacked by the spider mite *Tetranychus urticae* Koch*,* were less attractive to the predator *Phytoseiulus persimilis* Athias-Henriot, in comparison with the wild-type plants producing MeSa [41].

As already mentioned, the accession/cultivar-specific volatile blend can determine plant susceptibility or tolerance to insect pests. Raghava *et al.* [58] analysed the response of five tomato cultivars (PKM-1, Pusa Ruby, All Rounder, Lakshmi, Shaktiman) to both mechanical and *Spodoptera litura* Fab.-induced damage. Variations in both quantity and composition of VOCs emitted by the cultivars were correlated with different levels of attraction to beneficial parasitoids; in particular, the parasitoid *T. chilonis* was found to be more attracted by cultivars emitting higher amounts of β-phellandrene, *p*-cymene, carene, limonene, and caryophyllene (e.g. cv. Shaktiman).

Through molecular analysis and VOC profiling, D’Esposito *et al.* [59] investigated the underlying differences between a tolerant and a susceptible tomato genotype. These analyses revealed several genetic variances in enzymes involved in volatile biosynthesis. Volatilome profiling confirmed differences in VOC emission between the two cultivars with high emissions of γ-terpinene and δ3-carene in the tolerant plants, and a high level of camphene, α-phellandrene, eucalyptol, (*E*)-2-hexenal, and *cis*-3-hexenol in the susceptible ones. Kortbeek *et al.* [50], studying the differences among several tomato cultivars and accessions in resistance against *B. tabaci* and *Frankliniella occidentalis* (Pergande), highlighted the importance of intrinsic production of phytochemicals and VOCs stored in the trichomes to repel the pests. The role of VOCs as direct pest repellents in tomato has been the subject of various studies. For example, the attack of *Myzus persicae* (Sulzer) induced VOC emissions that repelled the whitefly *B. tabaci* [69], while VOCs emitted by drought-stressed tomato plants made these plants less attractive to *Tuta absoluta* (Meyrick) and *B. tabaci* than well-watered plants [70]. Recent findings suggest that increased phenylpropanoids/benzenoids production may be linked to the repulsion of the harmful insect *T. absoluta* [39].

Another crucial interaction between plants and insects mediated by VOCs involves those insects that are vectors of pathogens. Plant VOCs can indeed attract or repel vectors, thereby influencing disease transmission and spread. Mas *et al.* in 2014 [71] showed that the psyllid *Bactericera cockerelli* (Šulc) prefers to settle on healthy tomato plants when carrying the bacterium Candidatus *Liberibacter solanacearum*, but it prefers feeding on already infected plants when bacterium free. Similar findings have also been reported for viruses spread by the whitefly *B. tabaci*, where differences in the blend of VOCs emitted by infected and noninfected plants seem to attract the vector, contributing to the maximization of the pathogen spread (Luan *et al*. [72]: *B. tabaci/*tomato yellow leaf curl China virus TYLCCNV; Fereres *et al.* [73]: *B. tabaci*/tomato severe rugose virus ToSRV; Ghosh *et al.* [74]: *B. tabaci*/tomato yellow leaf curl virus TYLCV). How vectors can be more attracted to healthy plants when carrying pathogens is still unknown. An original hypothesis is that a modification in the expression of genes encoding odorant-binding proteins (OBPs) occurs in the insect once it becomes viruliferous, probably due to the direct action of the virus. This alteration might allow the vector to better identify VOCs emitted by healthy plants [75].

## Tomato VOC emissions in the interaction with pathogens

The role of VOCs in plant pathology has often been considered for potential applications, focusing on their direct antimicrobic actions, as well as indicators of plant responses to pathogens for early detection of infections. Plants can perceive the presence of a pathogen through pathogen-associated molecular patterns and activate a first line of immunity, known as pattern-triggered immunity (PTI). PTI responses involve the generation of reactive oxygen species (ROS), the activation of mitogen-activated protein kinase (MAPK) pathways, the alterations in intracellular calcium levels, and the production of a remodulation of immune-related genes expression. The overexpressed genes are generally involved in callose deposition in the cell wall, stomatal closure, and the synthesis of metabolites that suppress pathogen growth. Some pathogens can overcome this line of defence, delivering effectors into plant cells, virulence factors which stimulate a second line of plant defence, the effector-triggered immunity (ETI). Production of salicylic acid and activation of SAR, or hypersensitive response (HR) with programmed cell death that limits biotrophic pathogen spread, are the outcome of ETI [76]. In this complex crosstalk, several studies have proved that, besides the defence-related genes activated in pathogen’s response, the modulation of genes involved in VOCs biosynthesis (such as *TPSs*, *LOX*, *HPL*, *ADH*) also has a predominant role [36, 45, 77]. The direct effect of plant VOCs in the limitation of pathogen growth has been widely demonstrated, even if mainly in vitro [8, 9, 78, 79]. However, the elicitation of VOC production during pathogen infection may serve multiple purposes, such as directly disrupting the pathogen, preserving cellular components from oxidative damage, and spreading signals throughout the plant tissues [10].

When responding to necrotrophic pathogens, plants mainly activate the jasmonate (JA) signalling pathway, which is associated with strong emission of GLVs and some terpenes such as (*E*)-β-ocimene [80–82]. Conversely, the contact with biotrophic pathogens triggers the SA signalling pathway with HR response associated with SA derivatives methyl salicylate and salicylaldehyde, monoterpenes a-pinene, a-phellandrene, b-phellandrene, and limonene [36].

Hernández-Aparicio *et al.* [45] compared levels of resistance to *Fusarium oxysporum* f. sp. *lycopersici*, gene expression and VOC emission between two tomato isogenic lines, the resistant accession LA2828, and the susceptible accession LA3472. Pathogen infection resulted in the emission of methyl- and ethyl-salicylate (involved in SA and ABA signalling pathway) in the susceptible line, whereas in the resistant line, the infection caused emission of terpenoids (3-carene, (*Z*)-linalool oxide, dihydroactinidiolide, β-ionone, α-caryophyllene, and three isoforms of the sesquiterpene elemene) and of GLV derivatives of the LOX activity (JA signalling pathway). The resistant accession also consistently upregulated genes involved in the JA biosynthesis pathway, such as the allene oxide synthase (*AOS*) and *TomloxD* (*SlLOX4*), one of the described tomato 13-LOXs [45].

López-Gresa *et al.* [36] reported that tomato infection with avirulent strains of the bacterium *Pseudomonas syringae* pv. tomato was associated with the production of the GLV (*Z*)-3-hexenol and hydroxylated monoterpenes (e.g. linalool, α-terpineol, and 4-terpineol), and with the upregulation of *TomloxF* (*SlLOX6*) and *TPS5* genes, while virulent strains of the same bacterium elicited the activation of the SA pathway, necrosis, and consequent production of SA-derived VOCs. Besides their implication in JA signalling pathway activation, the emission of GLVs in response to pathogens seems to play multiple roles in plant protection [10, 83]. Direct involvement of GLVs in the regulation of stomatal closure to limit the spread of *P. syringae* pv tomato as part of the ETI response was reported [84]. Enhanced resistance to the phytopathogen fungus *Alternaria alternata* f. sp. *lycopersici* was also reported in tomato mutants constitutively expressing HPLs (involved in GLV synthesis) [85]. All these observations underline that GLV may not only be by-products of membrane degradation, as often assumed, but also activate immune responses after infection [80, 83, 86].

Trying to individuate markers for an early detection of the infection, many studies exploited VOCs emitted by the pathosystem tomato–*Botrytis cinerea* [80, 86, 87], which produce a strong response, with JA signalling activation and GLVs bursting within the first hour after infection [87]. However, in some instances, the activation of the SA pathway in response to *B. cinerea* infection was observed in tomato [86, 87], triggering a robust release of MeSa, the development of extensive necrosis, and production of the volatile TMTT (*E*,*E*)-4,8,12-trimethyl-1,3,7,11-tridecatetraene [81].

These few examples indicate the complexity of VOC involvement in plant–pathogen interactions, where VOCs’ role as elicitors and effectors is not always clarified.

## Tomato VOC emissions associated with abiotic stresses

Abiotic stresses involve alteration from optimal conditions of water availability, temperature, light, chemicals in the soil, ozone, mechanical damages, and many more environmental factors, resulting in a reduction of plant growth and yield. Plant response to abiotic stresses is complex and may involve the activation of several different pathways [88, 89]. One of the primary effects of abiotic stresses is that reduced photosynthesis is not able to use the electron transport generated by photosystems. The excess of electron transport causes the overproduction of ROS, which should be scavenged by enzymatic (e.g. enzymes active in the water–water cycle in chloroplasts) and nonenzymatic (e.g. carotenoids) plant antioxidant systems. If ROS are not scavenged by antioxidants, they can target Ca^2+^ membrane channels, altering the calcium ion fluxes and triggering complex downstream signalling cascades, or directly injure cell membranes (e.g. thylakoids), lipids, and proteins [90].

Terpenes, particularly isoprene, play a dual role in mitigating stress effects by acting as ROS scavengers and membrane stabilizers, thereby protecting cellular structures from oxidative damage and preserving membrane integrity under stress conditions [5, 91]. The enhanced production of terpenes, associated with the upregulation of genes related to terpenoid biosynthesis, mainly belonging to the TPS family, is part of the abiotic stress signal cascade [88] and has been extensively documented and correlated with plant tolerance to abiotic stresses [92].

The volatile emissions of tomato plants in response to abiotic stresses (salinity, flood, extreme temperatures or drought) have also been the subject of many studies. Heat stress, mild or severe, has been associated with an enhanced production of VOCs, especially in the initial stages of stress treatments [93]. In tomato plants, GLVs, isoprene, monoterpenes, and sesquiterpenes (especially 2-carene, limonene, α-phellandrene, β-phellandrene, α-humulene, and β-caryophyllene; Tables S2 and S3) have been reported as heat-induced VOCs [37, 94], possibly with genotype-dependent differences [93]. Interestingly, Pazouki *et al.* (2016) [37] identified three distinct phases in the response of tomato leaves exposed to heat spells of varying intensity: (i) immediate increase: upon exposure to heat stress, the emission of mono- and sesquiterpenes increased significantly; (ii) transient decline (2–10 hours): after the initial spike, VOC emissions dropped below control levels within 2 to 10 hours of recovery. (iii) late recovery (24 hours): VOC emissions rose again after 24 hours, indicating a delayed response after the stress had subsided. However, this is true for mild heat spells, but high-intensity stress (*T* > 46°C) causes permanent damage to the plants with no recovery of photosynthetic functions [95].

Low temperatures also seem to induce VOC emission. This is somehow counterintuitive, as volatilization of VOCs depends on temperature and should be reduced in cold environments. However, as in the case of high temperatures, massive emissions of GLVs may reflect the breakdown of fatty acids in cell membranes during cold stress [94].

Salinity is also correlated [96] with an increase in terpene production in tomato plants, especially (*Z*)-β-ocimene, 2-carene, and β-phellandrene, in five tested cultivars (Cherestur, Cheglevici, Dolat, Rudna, and Giera).

During water deficiency episodes, VOC emissions are generally influenced by two contrasting factors: (i) stress-induced de novo terpenoids biosynthesis and (ii) increased resistance to VOC emissions due to stomatal closure. These two contrasting factors may lead to either an increase or a decrease in total VOC emissions, often depending on genetic background or on the severity of the stress [97]. De novo biosynthesis often involves VOCs helping plants cope with drought, such as antioxidant volatiles (monoterpenes and sesquiterpenes) and membrane stabilizers (isoprene) [95, 98, 99]. Induced VOCs have emission rates often directly proportional to the severity of the stress [37, 100], especially for those that are independent of stomatal control (e.g. isoprene). Total VOC changes during drought stress have been correlated with modifications in plant indirect defence, with an enhanced attraction for some insect pests, e.g. *T. absoluta* [70] and/or a reduced attraction for beneficial organisms like *Microplitis croceipes* and *Podisus maculiventris* [101]. These findings emphasize how different stress factors can interact, accumulate, and have a combined effect on plant health. Interestingly, VOC emission in drought-stressed plants is sustained even when photosynthesis is totally suppressed [102], indicating activation of biosynthetic pathways alternative to the use of photosynthetic intermediates [92].

The emission of VOCs by tomato cultivars under drought stress has recently been studied to determine whether VOCs could serve as indicators of water status in field cultivation. This approach could provide a cost-effective tool in crop water management for field monitoring and support decision-making systems. Sensors for isoprene, ethylene, ethanol, methanol, and acetaldehyde were tested, yielding promising results in the case of ethylene sensors [103]. Additionally, a sensor for the sesquiterpene β-caryophyllene, another putative water stress marker, has recently been developed [104].

## The role of VOCs in plant–plant communication in tomato

**Figure 4:**
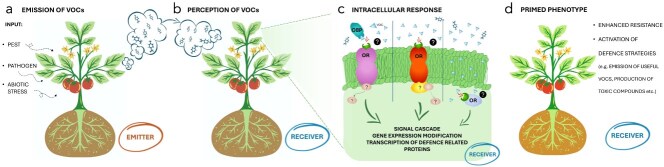
Schematic representation of plant-to-plant communication mediated by VOCs. (a) In response to external stimuli such as pest or pathogen attack and abiotic stress, an emitter plant releases VOCs. (b) These VOCs are perceived by a neighbouring (receiver) plant. (c) Upon perception, VOCs enter the leaf, either directly or thanks to OBPs and/or ORs, and trigger a signal transduction cascade that leads to gene expression reprogramming and activation of defence-related pathways. (d) As a result, the receiver plant acquires a *primed phenotype*, characterized by enhanced resistance and the activation of defensive strategies, such as the emission of its own VOCs or the production of toxic compounds.

In the last four decades, the possible roles and potential applications of VOCs in plant–plant communication have been repeatedly documented [2, 19, 25]. VOC-based plant–plant communication involves a series of events that generally follow a specific sequence. First, emitting plants (emitters) must release a VOC blend capable of reaching neighbouring receiving plants (receivers). Second, the receivers must perceive the VOCs. Third, perception must trigger a series of changes that generally ‘prime’ receivers, inducing alterations in their metabolism [4] (Fig. 4).

Confirming plant–plant communication, the work of Runyon *et al.* [105] showed that VOCs emitted by tomato plants are perceived by nearby dodder plants (*Cuscuta pentagona* Engelm), which use them as a cue to locate the host plant. While it is assumed that VOCs can reach the mesophyll mainly through the stomata, how the lipophilic molecules overcome the aqueous cell wall region to reach the cell membrane is currently debated [106]. Researchers are trying to discover whether plants own a sensing system similar to the one developed by animals, with nonspecific lipid transfer proteins acting as OBPs, which vehicle the molecules on odorant receptor (OR), or perceive odours without specific OBPs or receptors [5, 22]. Four presumed OBPs have been described in plants, binding MeJA [107], MeSa [108], and sesquiterpenes [22, 109]. However, it cannot be excluded that plants indeed perceive VOCs following other mechanisms. For example, the lipophilic nature of many bioactive plant VOCs lets us speculate that VOCs might dissolve in the membrane and influence its permeability to ions (Ca^2+^ and K^+^), modifying the membrane potentials and triggering the signal cascade. Zebelo *et al.* [110] showed changes in Ca^2+^ fluxes in tomato cells after exposure to VOCs emitted by plants under *S. littoralis* caterpillars feeding. The effects of VOCs on the cell membrane electric potential were also confirmed in tests with synthetic VOCs (α-pinene, β-caryophyllene, and GLVs like (*Z*)-3-hexenal, (*E*)-2-hexenal, and (*Z*)-3-hexenyl acetate), chosen among those induced by *S. littoralis* attack. Recently, these results have also been confirmed in a study on *Arabidopsis thaliana* [111].

While the mechanism at the basis of VOC perception by receiving plants is slowly coming to light, many studies have proved that foreign VOCs, emitted by stressed plants, may prime defensive responses in receivers, starting with the modulation of gene expression [112, 113] and ending with production of secondary metabolites active against forthcoming biotic or abiotic stresses [114–116]. All defensive responses may directly protect against stressors or may indirectly call for other protective organisms. For example, tomato plants primed via exposure to plants infested by *T. vaporariorum* increased emission of the sesquiterpenes α- and β-caryophyllene, and of MeSa, with a consequently enhanced resistance to the pathogen *Pseudomonas siringae* pv. tomato, as shown by measurements of pathogen spread and *PR-1* and *GLU* (encoding for acidic β-1,3-glucanase) gene expression [20]. On the other hand, the activation of indirect defence (the capacity of tomato plants to attract parasitoids) was observed in receiver plants primed with VOCs (the monoterpenes α-pinene, camphene, and methyl salicylate) emitted by donor plants subjected to aphid infestation and/or water stress [23] (Tables S2–S4). Another study used the tobacco mutant line NtOS2, overexpressing β-ocimene synthase gene (*PlOS),* as a VOC emitter. β-Ocimene indeed elicited direct and indirect (parasitoid attraction) resistance against aphids in receiver tomato plants [65].

**Figure 5:**
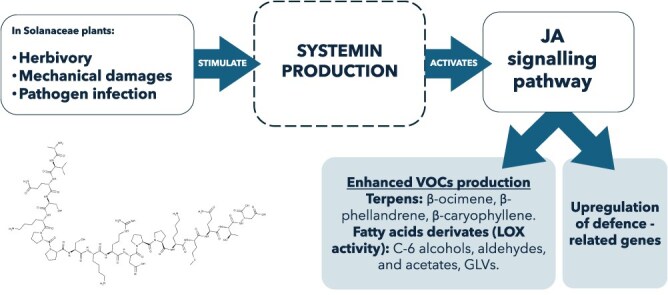
Systemin-mediated activation of plant defence responses in Solanaceae: herbivory, mechanical damage, and pathogen infection stimulate systemin production; systemin activates the jasmonic acid (JA) signalling pathway, leading to the upregulation of defence-related genes and the enhanced production of VOCs; these VOCs include terpenes (e.g. β-ocimene, β-phellandrene, β-caryophyllene) and fatty acid derivatives produced via lipoxygenase (LOX) activity, such as C6 alcohols, aldehydes, acetates, and green leaf volatiles (GLVs).

VOC emissions in tomato have been linked to the synthesis of systemin, a small plant peptide hormone typical of the Solanaceae family, produced to spread an alarm signal within the plant mainly after herbivory attack (Fig. 5). Once synthesized after injury, or provided to the tomato plant under experimental conditions, systemin [117] triggers a signalling cascade that leads to the release of linolenic acid, a precursor of JA [118], as well as to upregulation of *SlLOX* and *AOS* genes, and to the emission of bioactive VOCs that attract predators and parasitoids of insect pests [18, 41, 46, 60, 119–121].

Numerous studies have also explored the impact of systemin on plant-to-plant communication. Tomato mutants overexpressing the systemin precursor prosystemin, with constitutive activation of the systemin–JA pathway, have been used as VOC emitters in priming tests. VOCs emitted by these plants can influence neighbouring plants by altering their transcriptome, eliciting the expression of genes related to plant response and enhancing both direct and indirect defences against herbivores. This finding highlights the potential of systemin-induced VOCs in orchestrating interplant signalling and defence strategies [60]. Zhang *et al.* [18] showed that the priming effects can be cultivar dependent, using two different cultivars of tomato (Moneymaker and Castlemart) and *Prosystemin*-overexpressing mutants (*35S::prosys* Castlemart).

## Can VOCs be used as a tool for protecting tomato crops?

The data reviewed here highlight, first and foremost, that the ability to produce large quantities of specific VOCs, particularly terpenes, significantly enhances tomato plant resistance to pest attacks. Beyond pest resistance, VOCs are also involved in the plant responses to pathogens, either by triggering mechanisms like stomatal closure or by inducing pathogen resistance.

Tomato plants exhibit a remarkable ability to respond to biotic stresses by activating different mechanisms, mediated by either the salicylate or jasmonate signalling pathways [80, 86, 87]. Table 1 lists the most important VOCs emitted by tomato plants in response to stresses.

**Table 1:** Most important VOCs emitted by tomato plants and their role.

	**VOC**	**Role**	**Authors**
**Insects**	Β-Phellandrene	*T. chilonis p*arasitoid attraction	Raghava *et al.* [58]
	*p*-Cymene		
	Carene		
	Limonene		
	Caryophyllene		
	(8*S*9*R*)-(*E*)-Caryophyllene	*A. ervii* parasitoid attracti*on*	Sasso *et al.* [63]
	Methyl-salicylate		
	(*Z*)-3-Hexen-1-Ol		
	3-Carene	*E. formosa* parasitoid attraction	Ayelo *et al.* [67]
	β-Ocimene		
	β-Myrcene		
	α-Phellandrene		
	α-Pinene	*N. tenuis* predator attraction	Ayelo *et al.* [68]
	3-Carene		
	β-Ocimene		
	α-Phellandrene		
	Curcumene	Confer *B. tabaci* resistance	Bleeker *et al*. [47]
	Zingiberene		
	γ-Terpinene		
	*p*-Cymene		
	γ-Terpinene	Confer *T. absoluta* resistance	D’Esposito *et al.* [59]
	δ-3-Carene		
	Camphene	Confer *T. absoluta* susceptibility	D’Esposito *et al.* [59]
	α-Phellandrene		
	Eucalyptol		
	(*E*)-2-Hexenal		
	(*E*)-3-Hexenol		
**Abiotic stress**	2-Carene	Indicate heat stress	Copolovici *et al.* [94], Pazouki *et al.* [37]
	α-Phellandrene		
	Limonene		
	β-Phellandrene		
	α-Humulene		
	β-Caryophyllene		
	GLVs		
	GLVs	Indicate cold stress	Copolovici *et al.* [94]
	(*Z*)-β-Ocimene	Indicate salinity stress	Tomescu *et al.* [96]
	2-Carene		
	β-Phellandrene		
	2-Carene		
	α-Phellandrene		
	Limonene		
	α-Humulene		
	β-Caryophyllene		
	*Monoterpenes*	Indicate drought stress	Catola *et al.* [23], Pagadala Damodaram *et al.* [70]
	*Sesquiterpenes*		
**Pathogens**	β-Caryophyllene	Inhibit *P. infestans* growth	Arafa *et al.* [52]
	Isocurcumenol		
	Ledene oxide		
	Geranyl-α-terpinene		
	9-*cis*-Retinoic acid		
	GLVs, hexenyl derivatives	Indicate *B. cinerea* infection, activation of response via jasmonate pathway	Jansen *et al.* [86]
	(*E*)-β-Ocimene		
	TMTT-(*E*,*E*)-4,8,12-trimethyl-1,3,7,11-tridecatetraene	Indicates *B. cinerea* infection, activation of response via salicylate (HR) pathway	Jansen *et al.* [87], Kasal-Slavik [80]
	3-Carene	Emitted after *F. oxysporum* infection in resistant plants	Hernández-Aparicio *et al*. [45]
	(*Z*)-Linalool oxide		
	Dihydroactinidiolide		
	β-Ionone		
	α-Caryophyllene		
	Elemene (β, γ, δ)		
	Methyl-salicylate	Indicate *F. oxysporum* infection in susceptible plants]	Hernández-Aparicio *et al.* [45]
	Ethyl-salicylate		
	GLVs	Induce stomatal closure after *P. syringae* infection	López-Gresa *et al.* [36]

Detailed information is reported in Tables S2–S4.

The growing demand for innovative plant protection solutions, driven by the exigence of replacing conventional synthetic pesticides to sustainably control crop pests, has led to increased interest in using VOCs. With crop pests accounting for 25% of global crop losses [122], these alternatives are crucial within an Integrated Pest Management (IPM) framework. Exploiting the potential of VOCs in agricultural systems will enhance sustainable pest management, improve crop resilience to environmental stress, and reduce reliance on synthetic pesticides while promoting eco-friendly farming practices [123, 124].

Although the use of VOC-mediated plant defence to boost plant resistance has gained significant interest over the past 20 years [6, 26, 123, 125], it has not yet been fully implemented in practical crop protection. However, promising strategies within IPM are emerging and have already been tested, mainly in greenhouse conditions, such as the use of intercropping plants or association approaches (trap plants, companion sentinel plants, push–pull systems, priming inducer plants), and the application of synthetic VOCs and plant gene editing to enhance or suppress the production of specific VOCs. Since this review is focused on tomato cultivation, only examples related to this crop, in greenhouse or open field, will be reported. Examples considering other different species are too numerous and fall outside the scope of this work.

### Intercropping plants or association approaches

Most published studies on the effects of intercropping attributable to the action of VOCs are focused on pest management, whereas studies investigating their effects on plant pathogens are comparatively limited, though increasing especially at laboratory scale [126]. Intercropping of tomato with coriander (*Coriandrum sativum*) has been shown to reduce the incidence and the damage caused by the pest *B. tabaci*, achieving results comparable to synthetic insecticides [127], while also providing additional income from coriander sales [128]. Togni *et al.* [127] investigated the role of the constitutive volatiles released by coriander on *B. tabaci* behaviour, demonstrating that these volatiles reduce the attractiveness of this pest to tomato plants. This effect is not due to a repellent action or to a masking of tomato odours, rather due to VOCs interfering with host plant selection by *B. tabaci*. Greenhouse experiments confirmed that *B. tabaci* populations were higher in tomato monocultures compared to tomato–coriander intercrops. In Brazil, this practice has been adapted for organic tomato farming to control both *B. tabaci* and *T. absoluta* through combined bottom-up (plant-based) and top-down (natural enemy-based) mechanisms [129, 130]. Similar results were obtained by Padala *et al.* [131]. Olfactometer bioassays, electrophysiological tests and field experiments, showed that both dill (*Anethum graveolens* L.) and coriander have effects on *B. tabaci*, resulting in a lower whitefly number in plots with both plants.

In a large-scale greenhouse experiment, reduced whitefly populations were observed on French marigold (*Tagetes patula*) intercropped tomato due to the repellent effect of limonene, a major VOC produced by marigold flowers [132]. In a second experiment in the greenhouse [132], the potential for marigold plants and limonene dispensers to control a heavy infestation of whiteflies on tomato was again proved. In a recent experiment with potted tomato plants, it was demonstrated that VOCs released by potato onion (*Allium cepa* var. *agrogatum* Don.) plants, particularly dipropyl disulphide, alter the performance of tomato plants via modulation of the rhizosphere microbiota, specifically attracting beneficial bacteria [133].

An evolution of intercropping that also relies on the ecological function of plant VOCs in the agro-ecosystem is the ‘push–pull’ strategy. This is based on the interaction between trap plants and repellent plants, in relation to a specific insect. Although several studies have investigated push–pull strategies for the management of phytopathogens and pests in tomato crops, only a few have examined the mechanistic role of VOCs in mediating these interactions.

Lee and colleagues [134] developed an integrated push–pull strategy to manage *B. tabaci* in tomato greenhouses. Their approach combined yellow sticky traps and 520-nm green LED light, along with methyl isonicotinate as an attractant (pull), and 450 + 660-nm LED light, carvacrol, and ocimene as repellents (push). Moreover, geranium plants (*Pelargonium inquinans*) were also used as effective companion plants to repel whiteflies, while buckwheat (*Fagopyrum esculentum* Moench) was used as a banker plant to support populations of the beneficial predator *Cyrtopeltis tenuis*. This integrated system reduced whitefly density by over 68% after 50 days and led to a 143% increase in tomato yield compared to untreated controls. Mangrio [135] evaluated the effect of spearmint (*Mentha spicata*) and garden cress (*Lepidium sativum*) as effective repellent and attractant species, respectively, under field conditions, and associated the effects with the emission of specific VOCs, as previously suggested in literature [136, 137].

Success of intercropping is not always straightforward, as VOCs could be responsible for induction of priming responses or exert a direct effect on pests and pathogens. Moreover, different authors highlight how the success of a companion plant approach is context dependent, relying on precise combinations of plant chemistry, environment, pest type, and implementation method [138].

### Exogenous application of VOCs

Exogenous application of VOCs to boost plant resistance and resilience to diverse stresses is currently one of the most promising areas of VOC applied research. Many studies highlighted VOCs’ ability to pre-activate a defensive response in healthy plants [26, 124], leading to speculation about their potential as a new category of crop ‘bio-stimulants’.

Recent studies have demonstrated the success of continuous application of (*Z*)-3-hexenyl propanoate. When regularly dispensed to tomato plants in commercial greenhouses, *Z*-3-HP kept plant defences activated for over 2 months, reducing infestations of pests such as *T. urticae* and *T. absoluta* [139]*,* the root-knot nematode *Meloidogyne* spp. [140], and *N. tenuis* [141]. The application of β-caryophyllene and β-myrcene via dispensers increased the attraction of the wasp *E. formosa*, a *B. tabaci* parasitoid, improving pest management efficiency [142]. Similarly, limonene, when applied through a dispenser, acted as both a repellent and a plant defence elicitor, effectively controlling *T. vaporariorum* on tomatoes [143]. In a greenhouse experiment with (*Z*)-3-hexenol (z3HOL), Yang *et al.* [144] demonstrated induction of defence-related genes and HIPVs in tomato, resulting in a decrease in *B. tabaci* infestation and improvement of *E. formosa* parasitoid recruitment.

It is interesting to highlight how plant priming not only activates the JA and SA pathways but also enhances secondary metabolism, promoting the accumulation of compounds such as polyphenols, flavonoids, carotenoids, and antioxidant enzymes, in tissues like leaves and fruits, which contribute to food quality and offer protective effects in human cells [145, 146].

Exogenous VOC applications on tomato plants have been delivered also to improve responses to abiotic stress [147].

Many questions remain unanswered, such as the required amount of VOCs and the most efficient timing of their administration to effectively bolster plant defences. This is particularly relevant given the significant differences between controlled laboratory conditions and open-field environments where background air composition may confuse VOC signals. There is also a lack of information regarding the stability of VOCs in the atmosphere and the long-term effects of such priming on plant health and productivity. Using synthetic VOCs has some advantages over naturally occurring VOCs as their dose and application frequency can be better controlled. This has potential for development of VOCs as IPM tools, provided the associated cost will be economically sustainable.

### Genome editing

Thanks to advances in the omics field, new information has been acquired on the genes involved in VOC biosynthetic pathways [3, 148]. This has enabled genetic modifications in crops to enhance the emission of specific volatile compounds for plant’s defensive functions [124, 149–151]. Although tomato has been extensively studied as a target for genetic engineering [152, 153], most studies in this area have been conducted under laboratory and greenhouse conditions using model plant species such as *A. thaliana* or *Nicotiana tabacum*, rather than horticultural crops. Only a limited number of studies have reported transgenic tomato lines engineered to overexpress or suppress genes involved in VOC biosynthesis, mostly focusing on monoterpenes such as β-ocimene [65] and sesquiterpenes like β-caryophyllene [154].This may reflect the inherent complexity of manipulating plant metabolic networks, as modifying the VOC biosynthetic route can inadvertently influence other interconnected metabolic processes [155]. Beyond the technical aspects, the use of gene-edited plants presents additional challenges, as there are regulatory, ecological, and public perception issues to consider.

### VOCs as phenotyping markers of early plant stress detection

Precision agriculture integrates advanced technologies to enhance efficiency, productivity, and sustainability of farming systems. IoT (Internet of Things), Big Data, AI (artificial intelligence), and machine learning are central to this field, with sensors playing a crucial role in real-time crop monitoring and in enabling data-driven decisions to optimize growth and reduce biotic and abiotic stress impacts. In this context, monitoring VOC emissions from tomato plants, both in greenhouses and open fields [103, 104], could provide a valuable tool for enabling early detection of stresses such as water deficit and heat spells, and for planning timely interventions, optimizing water use, and minimizing crop losses [156, 157]. Array-based sensors, such as electronic (e-noses) and chemical noses, based on high-throughput and ultra-sensitive technologies (e.g. proton transfer reaction – mass spectrometry) have been developed to allow continuous and noninvasive monitoring of VOCs, substituting traditional and low-throughput VOC detection devices such as off-line GC–MS [158].

With specific reference to tomato applications, Li and colleagues [159] developed a low-cost, portable platform for the early, noninvasive detection of *P. infestans* (late blight) in tomatoes. The platform can detect key plant volatiles, such as (*E*)-2-hexenal, at sub-ppm levels within 1 minute, and uses pattern recognition techniques like principal component analysis to differentiate between healthy and infected plants. The authors demonstrated the ability of this system to detect infection as early as 2 days after inoculation, well before visible symptom appearance, with a diagnostic accuracy greater than 95%. The same authors developed another platform even more versatile, with the use of a wearable flexible sensor on tomato plants [160].

For what concerns the e-nose applications in tomato crop horticulture, many studies have focused on the development of systems able to determine fruit quality [161], maturity [162], or shelf life [163], consistently achieving excellent results regarding the ability of these instruments to discriminate volatile fingerprints of the desired categories. Application of e-nose in plant protection was attempted by Cui and colleagues [164] who correctly identified infection of *B. tabaci* in greenhouses or mechanical damage at very early stages. E-noses are specifically used for assessing plant mechanical damage, which is particularly useful for optimizing plant management in pre- and postharvest stages. Sun *et al.* [165] discriminated tomato seedlings with varying severities of mechanical damage using the e-nose system PEN2 (Airsense Analytics GmbH, Schwerin, Germany), equipped with 10 different metal oxide semiconductors. The same sensor (PEN2) correctly discriminated tomato plants infected with the pathogens *B. cinerea* and *Alternaria solani* [166]. A portable and low-cost e-nose was developed by the Digital Agriculture Food and Wine Group from the University of Melbourne (DAFW-UoM), discriminating the presence of soil-borne pathogens such as *F. oxysporum* and identifying inoculated soils, as demonstrated by Feng *et al.* [167]. Although sensors for monitoring plant-emitted VOCs show promising potential for development, their stability and selectivity in responding to various stressors remain subjects of ongoing investigation, especially in field conditions.

## Conclusions

The tomato plant produces a wide variety of VOCs, with more than 200 constitutive or stress-induced compounds very well investigated. Findings demonstrated that these VOCs are not mere by-products of plant metabolism; rather, many of them play active roles in interactions with other organisms, triggering plant defences against abiotic and biotic stressors, and spreading alarm signals that serve as cues for plant-to-plant communication. Despite challenges such as low VOC emission rates, or difficulties in achieving accurate quantification due to sampling and handling issues (i.e. rough handling of tomato leaves could cause trichome rupture), significant progress in VOC studies have been reached. It will be the task of future research to further deepen our understanding of the roles these molecules play in the physiology and ecology of tomato plants. Future agriculture will need to become increasingly sustainable, and to achieve this goal, it will be necessary to apply new biotechnologies. Among these, the use of VOCs to enhance plant resilience to various stresses is certainly one of the most promising.

## Supplementary Material

Web_Material_uhaf181
